# High‐Resolution MRI Revealed Different Etiology‐Specific Associations With Cerebral Infarction in Adult Moyamoya Vasculopathy

**DOI:** 10.1002/acn3.70380

**Published:** 2026-04-01

**Authors:** Guangsong Han, Jiachun Pan, Yuehui Hong, Xiaoyuan Fan, Ming Yao, Lixin Zhou, Yicheng Zhu, Feng Feng, Jun Ni

**Affiliations:** ^1^ Department of Neurology, State Key Laboratory of Complex Severe and Rare Diseases Peking Union Medical College Hospital, Chinese Academy of Medical Sciences and Peking Union Medical College Beijing China; ^2^ Department of Radiology, State Key Laboratory of Complex Severe and Rare Diseases Peking Union Medical College Hospital, Chinese Academy of Medical Sciences and Peking Union Medical College Beijing China

**Keywords:** cerebral infarction, high‐resolution MRI, moyamoya disease, moyamoya syndrome, moyamoya vasculopathy

## Abstract

**Objective:**

High‐resolution MRI enables detailed assessment of intracranial vessel wall pathology in moyamoya vasculopathy. We aimed to classify adult moyamoya vasculopathy etiologies using high‐resolution MRI and to examine subtype‐specific associations between high‐resolution MRI features and ischemic infarction.

**Methods:**

We enrolled 398 adult patients with moyamoya vasculopathy. According to high‐resolution MRI characteristics, 340 patients were classified as moyamoya disease (*n* = 279) or atherosclerosis‐associated moyamoya vasculopathy (*n* = 61). Hemisphere‐level analyses were performed using generalized estimating equations logistic models with patients as clusters to evaluate associations between high‐resolution MRI measures and ischemic hemispheres.

**Results:**

Compared with atherosclerosis‐associated moyamoya vasculopathy, moyamoya disease patients were more often female and more likely to have a family history of early‐onset stroke and posterior cerebral artery involvement. In the moyamoya disease group, greater middle cerebral artery stenosis, higher Suzuki stage, and more severe internal carotid artery, middle cerebral artery, and posterior cerebral artery involvement were independently associated with ischemic hemispheres, whereas a higher middle cerebral artery remodeling index was inversely associated with ischemia. In the atherosclerosis‐associated moyamoya vasculopathy group, higher middle cerebral artery remodeling index and more severe anterior cerebral artery involvement were independently associated with ischemic hemispheres, while other high‐resolution MRI metrics showed no significant associations.

**Interpretation:**

High‐resolution MRI‐derived quantitative vascular metrics show distinct ischemia‐related patterns in moyamoya disease and atherosclerosis‐associated moyamoya vasculopathy. Global steno‐occlusive burden with impaired middle cerebral artery remodeling characterizes ischemic hemispheres in moyamoya disease, whereas adverse middle cerebral artery remodeling and anterior cerebral artery involvement are key correlates of ischemia in atherosclerosis‐associated moyamoya vasculopathy.

## 1 Introduction

With the advancement of cerebrovascular imaging techniques and the deepening understanding of cerebrovascular pathology, the concept of moyamoya vasculopathy (MMV) has gradually emerged in our field of vision, which encompasses the characteristic vascular abnormalities of progressive chronic stenosis or occlusion at the circle of Willis accompanied by moyamoya collateral vessel formation, irrespective of whether it occurs as moyamoya disease (MMD) or moyamoya syndrome (MMS) associated with specific underlying disorders or risk factors [1]. The increasing diagnosis of MMV in adults, along with the higher detection of asymptomatic cases in recent years, indicates that MMV remains an underrecognized or misdiagnosed cause of stroke [2].

Although patients with MMS typically present with symptoms of ischemic stroke or intracranial hemorrhage, similar to those with MMD, the former demonstrate less development of moyamoya vessels and are more likely to exhibit unilateral involvement [3]. The application of high‐resolution MRI (HRMRI) in MMV in recent years has facilitated the diagnosis of MMD when combined with clinical history, particularly in distinguishing MMD from atherosclerosis‐associated moyamoya vasculopathy (AS‐MMV) [4]. The characteristic manifestations of MMD on HRMRI vascular wall imaging can be summarized as follows: vascular collapse, reduced outer diameter, concentric thickening, constrictive/negative remodeling, and more deep vascular flow voids. In contrast, AS‐MMV demonstrates more obvious atherosclerotic plaque burden, eccentric thickening and expansive/positive remodeling, accompanied by less deep vascular flow voids [5, 6, 7, 8]. Some HRMRI features are also considered to be related to the long‐term outcome of MMD, such as vessel wall contrast enhancement [9, 10]. These findings demonstrate the significant value of HRMRI in the etiological diagnosis of MMV and stroke risk prediction.

Previous epidemiological studies have shown that the annual incidence of cerebrovascular events in adults with asymptomatic MMD under conservative management is approximately 2.4%–5.7%, with hypertension, hyperlipidemia, and disturbed cerebral hemodynamics at initial diagnosis identified as risk factors [2, 11, 12]. However, there remains a lack of noninvasive and clinically practical methods to assess intracranial vascular compensatory capacity in MMV patients and predict stroke risk, which is critical for guiding surgical timing and clinical treatment strategies. Therefore, HRMRI could be utilized as an effective imaging modality to identify differences between asymptomatic and symptomatic MMV patients, which may help elucidate the risk factors for cerebrovascular events in MMV.

In our study, we conducted 3.0 T HR‐MRI in a large prospective cohort of adult MMV patients and established diagnoses of adult MMD and AS‐MMV based on HRMRI characteristics. Subsequently, we performed comparative analyses between asymptomatic and ischemic MMV hemispheres within MMD and AS‐MMV groups respectively, evaluating severity of vascular involvement and vessel wall imaging characteristics. These investigations aimed to identify etiology‐specific associations with ischemic stroke in MMV, thereby providing guidance for early diagnostic strategies and personalized therapeutic interventions.

## 2 Materials and Methods

### 2.1 Study Participants

All patients included in this study were consecutively enrolled from our registry cohort entitled “clinical cohort research of moyamoya vasculopathy” from September 2017 to April 2025. Patients meeting all of the following criteria were enrolled: 1. Age over 18 years old; 2. Radiologically confirmed MMV diagnosis based on MRI/MRA findings [13], requiring: (a) stenosis or occlusion at the circle of Willis: the terminal intracranial internal carotid artery (ICA) and/or proximal segments of the anterior cerebral artery (ACA) and/or middle cerebral artery (MCA); (b) evidence of collateral vascular networks demonstrated by either: abnormal basal ganglia and/or periventricular vascular networks on MRA, or ≥ 2 vascular flow voids in the basal ganglia and/or periventricular white matter on MRI. In addition, patients without apparent ICA involvement on MRA were included if they showed clear stenosis or occlusion of the ACA and/or MCA accompanied by typical moyamoya collateral vascular networks, that is, they fulfilled the 2012 MRA criteria [13] for MMD but no longer met the revised 2022 MRA criteria [14] requiring ICA involvement. Exclusion criteria comprised: (i) poor imaging quality; (ii) insufficient clinical documentation; (iii) complicated with systemic vasculopathies (e.g., infectious or systemic vasculitis and other diffuse systemic vascular disorders) or radiation‐induced vasculopathy. Data on clinical and demographic factors such as age, gender, clinical events related to MMV, blood glucose levels, risk factors of cerebral vascular diseases, and family history of early‐onset stroke (defined as stroke occurring in first‐degree relatives before age 60, including both ischemic and hemorrhagic stroke) were collected. None of the enrolled patients underwent direct or indirect extracranial–intracranial bypass surgery before or during the imaging evaluations; all patients were treated with medical management during the study period.

### 2.2 HRMRI Protocol

HRMRI of the intracranial vessel wall was performed in all patients using the same 3.0‐T MR scanner with a standardized acquisition protocol. By acquiring all examinations on a single 3.0‐T system, interscanner variability was minimized and vessel wall characteristics could be compared more reliably across patients. HRMRI was used to evaluate luminal stenosis, wall thickening, remodeling patterns, and the extent of involvement of major intracranial arteries, providing quantitative and qualitative information on vessel wall pathology in patients with MMV. The following MRI sequences were used for assessment: T1‐weighted imaging (T1WI); T2‐weighted imaging (T2WI); fluid‐attenuated inversion recovery (FLAIR); diffusion‐weighted imaging (DWI); time‐of‐flight MR angiography (TOF‐MRA); and high‐resolution MR (HRMRI). The parameters for the 3.0 T scanner were summarized in Table S1.

### 2.3 Image Assessments

#### 2.3.1 The Severity of Vascular Involvement

All enrolled MMV patients underwent standardized bilateral assessment of vasculopathy severity using the Suzuki staging system [15] (detailed in Table S2) and the MRA scoring system [16] (detailed in Table 1) on TOF MRA. Each cerebral hemisphere was independently evaluated to systematically quantify disease burden.

**TABLE 1:** MRA scoring system.

Findings	score
ICA	
normal or minimum equivocal change of the ICA	0
apparent stenosis at the terminal of ICA (distal to the PCoA)	1
discontinuity of the signal of the terminal of ICA	2
no depiction of the intracranial ICA	3
MCA	
normal or minimum equivocal change of the horizontal portion of the MCA	0
apparent stenosis of the horizontal portion of the MCA	1
discontinuity of signal of the horizontal portion of the MCA	2
no depiction of most of the MCA	3
ACA	
normal signal intensity of the A2 and its distal branches	0
signal decrease or loss of the A2 and its distal branches	1
no depiction of the ACA	2
PCA	
normal or equivocal stenotic change of the P2 and its distal branches	0
signal decrease or loss of P2 and its distal branches diminishes	1
no depiction of the PCA	2

Abbreviations: ACA, anterior cerebral artery; ICA, internal carotid artery; MCA, middle cerebral artery; PCA, posterior cerebral artery; PCoA, a posterior communicating artery.

#### 2.3.2 The Vessel Wall Image Assessments

Based on HRMRI sequence, vessel walls of M1 portion of MCA were independently evaluated at the site of maximal stenosis or just proximal to the occlusion on each cerebral hemisphere. The HRMRI evaluation data of the affected vessel wall are calculated relative to the normal “reference vessel.” Definition of reference vessel: (1) the nearest plaque‐free or morphologically normal vessel wall segment adjacent to the site of maximal stenosis; (2) If the entire M1 segment is affected, the corresponding plaque‐free segment of the contralateral M1 will serve as the reference vessel. The percentage degree of MCA stenosis was calculated as (1 − lumen area of MCA/reference vessel lumen area) × 100%. Eccentricity index (EI) was calculated as (maximum vessel wall thickness − minimum vessel wall thickness/maximum vessel wall thickness) × 100%. Concentric wall thickening was defined as EI < 50%, while eccentric wall thickening was classified as EI ≥ 50%. Remodeling index (RI) was the ratio of vessel area at MCA to the reference vessel area. Negative remodeling was defined as RI < 0.95, while positive remodeling was classified as RI > 1.05. If atherosclerotic plaque signal (appear as eccentric wall thickenings with heterogeneous signal intensity) was identified within the vascular lumen, its morphological characteristics including location and longitudinal extent were documented.

#### 2.3.3 The differentiating criteria between MMD and AS‐MMV based on HRMRI was established based on previous literature [4, 5, 6, 7, 8, 17, 18]

Diagnostic vessel wall criteria of MMD:

1. Concentric, uniform wall thickening;
2. Vessel negative remodeling:
3. Prominent basal ganglia flow voids (≥ 2 unilateral voids);
4. Absence of atherosclerotic plaque.

Diagnostic vessel wall criteria of AS‐MMV:

1. Irregular, eccentric stenosis often extending beyond terminal ICA;
2. Eccentric wall thickening with lipid‐rich/hemorrhagic plaques;
3. Vessel positive remodeling:
4. The evidence of atherosclerosis in extracranial arteries.

#### 2.3.4 Definition of Ischemic and Asymptomatic Hemispheres

We compared the differences in vessel wall characteristics on HRMRI between the ischemic hemispheres and asymptomatic hemispheres in patients with MMV. Thirteen patients with hemorrhagic hemispheres were excluded from the analysis due to an insufficient sample size. The ischemic hemisphere was defined by the presence of MCA territory‐related infarction (including both acute and chronic phases) on T1WI, T2WI, DWI, and FLAIR sequences. MCA territory‐related infarction was radiologically confirmed as ischemic lesions in: (i) the MCA territory or (ii) anterior cortical/posterior cortical/subcortical watershed zones. The asymptomatic hemisphere was defined as the absence of detectable cerebral infarction or hemorrhage lesions on these sequences.

#### 2.3.5 The Blinding Procedures, Adjudication Rules, and Reproducibility of Images Assessments

To reduce subjectivity and incorporation bias, the imaging evaluations were carried out in separate, partially blinded steps. First, two experienced readers classified each patient as MMD or AS‐MMV in a dedicated reading session; they were aware that all patients had MMV but were blinded to the hemisphere‐level ischemic/asymptomatic categorization and to the quantitative HRMRI metrics used in the statistical models. Second, in another session, the severity of vascular involvement (stenosis scores and Suzuki stage) and vessel wall characteristics were assessed by observers who were blinded to the etiologic labels. Third, hemisphere status (ischemic vs. asymptomatic) was determined independently by a stroke neurologist based on clinical presentation and parenchymal MRI, blinded to the detailed vessel wall assessments. Discrepancies in etiologic classification were resolved by consensus discussion. The intrarater agreements for the rating of the severity of vascular involvement and the vessel wall images were assessed on a random sample of 20 subjects at 1 month interval. The intraclass correlation coefficient analysis showed a good reliability (detailed in Table S3).

### 2.4 Statistical Analysis

Skewed distributions continuous variables were presented as median (IQR), and comparisons between groups were performed using the Mann–Whitney test; normally distributed continuous variables were expressed as mean ± standard deviation, and intergroup comparisons were conducted using the *t*‐test. Categorical variables were summarized using percentages and were analyzed by the chi‐squared test. When comparing vessel wall characteristics on HRMRI between ischemic and asymptomatic hemispheres, all analyses were performed at the hemispheric level, with the two hemispheres from the same patient treated as a cluster. Because outcomes from the two hemispheres within an individual are correlated, we used generalized estimating equations (GEE) with a binomial distribution and logit link to model the probability of an ischemic hemisphere, specifying the patient identifier as the clustering variable and an exchangeable working correlation structure. All hemispheres from patients with unilateral or bilateral MMV were included in the analyses. Hemispheres without angiographically visible vascular involvement were retained and coded with the lowest vascular scores, and served as internal controls within the GEE framework. The dependent variable was whether a given hemisphere was the ischemic hemisphere. Imaging predictors included the degree of MCA stenosis, MCA eccentricity index, MCA remodeling index, Suzuki stage, and segmental scores of the intracranial arteries. All imaging parameters were entered into the models as continuous variables, and odds ratios (ORs) were interpreted as the change in odds of a hemisphere being ischemic per 1‐unit increase in the corresponding imaging measure. To evaluate the association between each imaging parameter and ischemic hemisphere while controlling for vascular risk factors, we fitted separate multivariable GEE models for each imaging parameter. Every model was adjusted for age, sex, hypertension, diabetes mellitus, hyperlipidemia, smoking, alcohol consumption, and family history of early stroke. These covariates were selected based on clinical relevance and previous literature, and imaging predictors were modeled one at a time to avoid multicollinearity between highly correlated vascular imaging measures. Model results are presented as odds ratios (ORs) with 95% confidence intervals (95% CIs). A two‐sided *p* value < 0.05 was considered statistically significant. All analyses were performed using R (R Foundation for Statistical Computing, Vienna, Austria) with the geepack package for GEE estimation.

## 3 Results

### 3.1 Clinical Characteristics of MMD and AS‐MMV Diagnosed by HRMRI

This study enrolled 398 adult patients with MMV, including 257 females (64.6%), with a median age of 41 years. Based on the predefined HRMRI characteristics and diagnostic definitions, the 340 enrolled MMV patients were further classified into two groups: 279 patients were diagnosed with MMD (Figure 1) and 61 patients with AS‐MMV (Figure 2). Univariate analysis revealed that compared with AS‐MMV patients, MMD patients in the study had a higher proportion of females (*p* = 0.049) and were more likely to have a family history of early‐onset stroke (*p* = 0.034) and posterior cerebral artery (PCA) involvement (*p* = 0.036) (Table 2).

**FIGURE 1:**
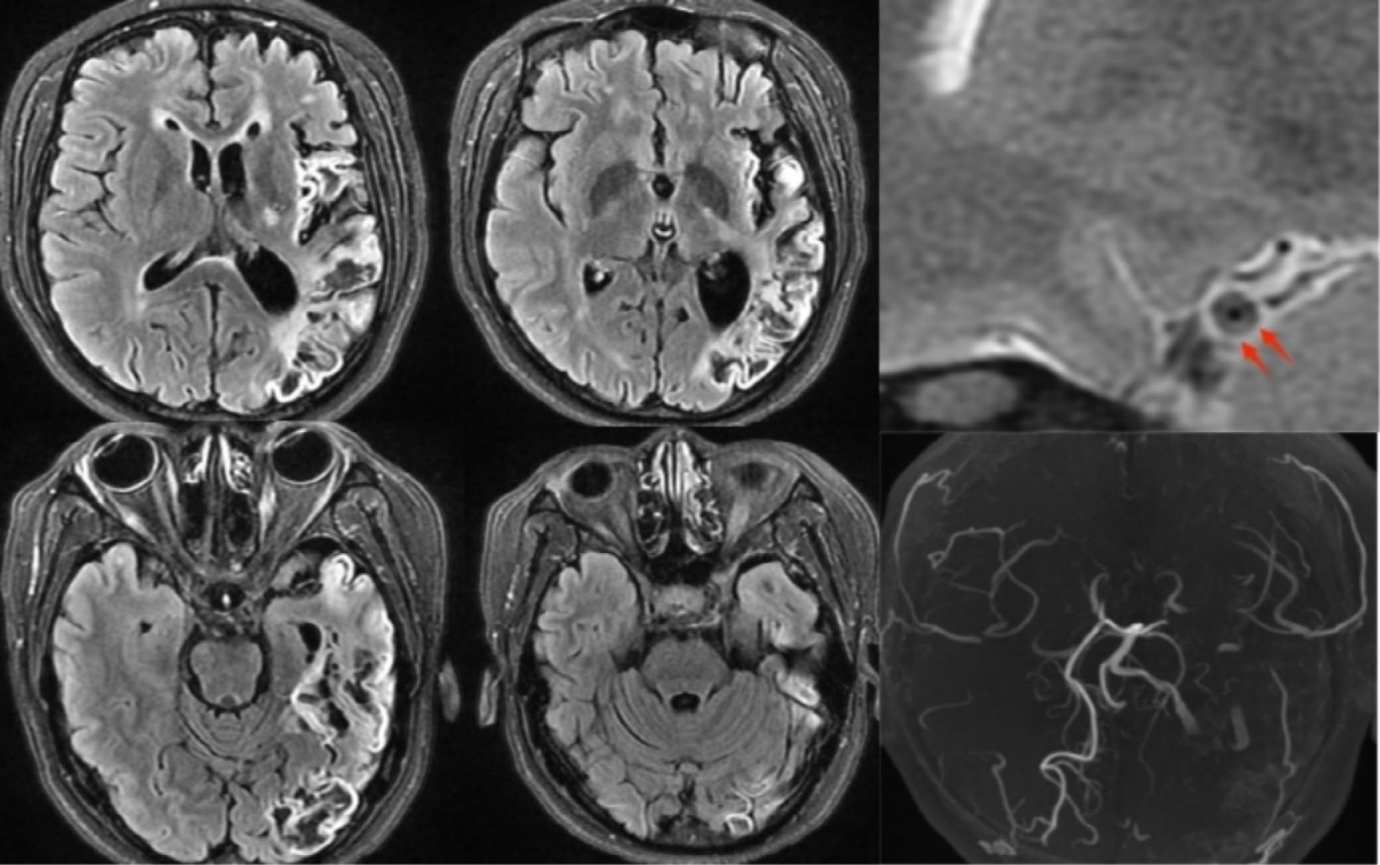
Fifty‐two‐year‐old male patient with MMD presented with imaging findings including: Left MCA territory infarction on FLAIR sequence. HRMRI showed concentric wall thickening and negative remodeling of the left middle cerebral artery (MCA) (red arrow). TOF‐MRA revealed signal loss in bilateral MCAs, bilateral anterior cerebral arteries and the terminal left internal carotid artery, along with signal discontinuity at the left posterior cerebral arteries (PCA) P2 segment, while the right PCA showed good visualization with compensatory dilatation.

**FIGURE 2:**
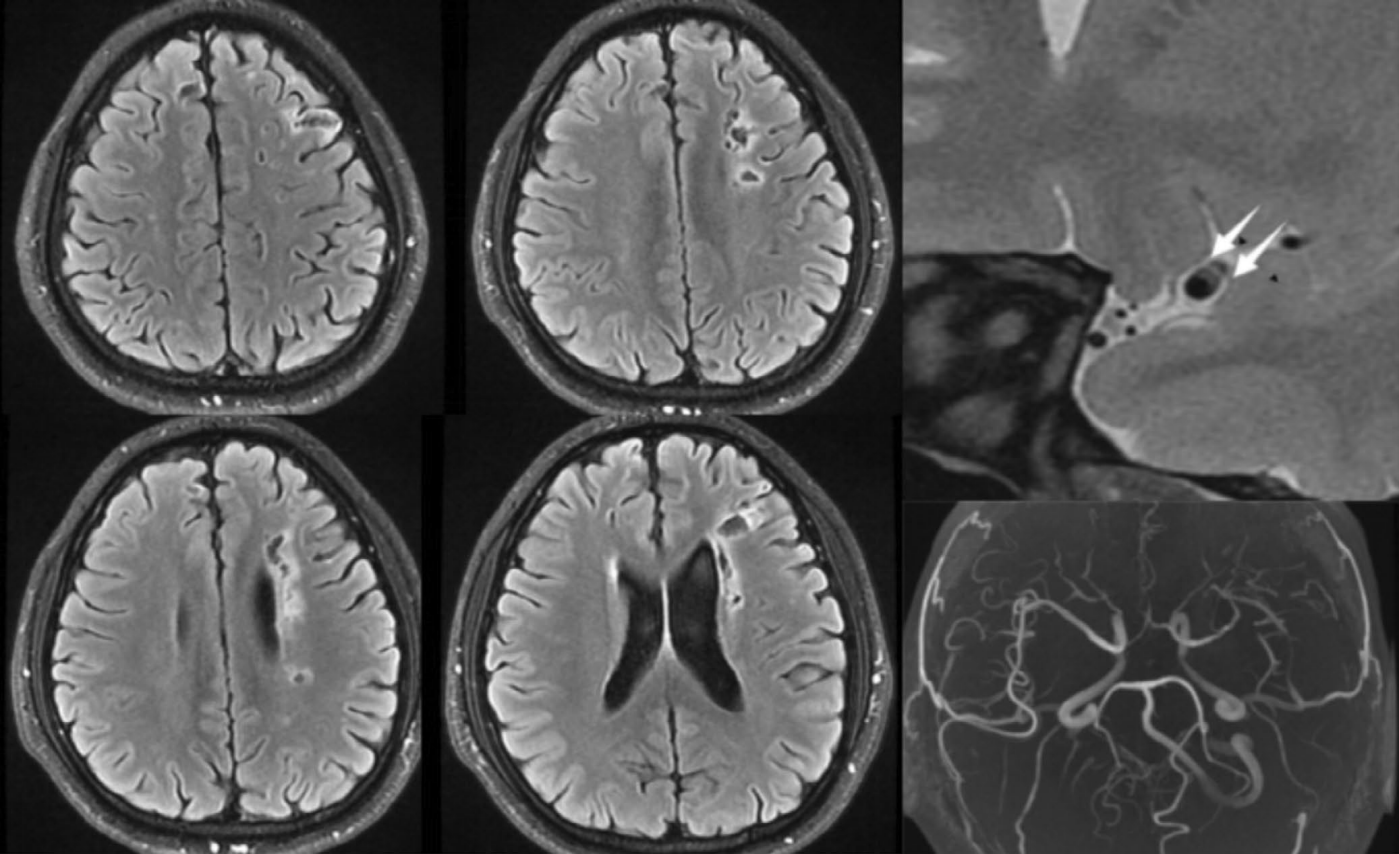
Twenty‐eight‐year‐old male patient with AS‐MMV presented with imaging findings including: Left watershed zone infarction on FLAIR sequence. HRMRI showed eccentric wall thickening, positive remodeling, and plaque signal in the left middle cerebral artery (MCA) (white arrow). TOF‐MRA revealed multiple focal stenoses in the left MCA along with signal decrease in bilateral anterior cerebral arteries, while both posterior cerebral arteries were well‐visualized.

**TABLE 2:** Comparison of clinical characteristics between MMD and AS‐MMV diagnosed by HRMRI.

	MMD (*n* = 279)	AS‐MMV (*n* = 61)	χ^2^/t	*p*
Gender(female)	188 (67.4%)	33 (54.1%)	3.883	0.049
Age	42.2 ± 12.9	42.0 ± 10.7	0.093	0.926
Hypertension	97 (34.8%)	17 (27.9%)	1.069	0.301
Diabetes mellitus	45 (16.1%)	6 (9.8%)	1.555	0.212
Blood glucose levels	5.56 ± 1.54	5.48 ± 1.67	0.408	0.683
Hyperlipidemia	53 (19.0%)	9 (14.8%)	0.604	0.437
Smoking	27 (9.7%)	7 (11.5%)	0.180	0.672
Alcohol consumption	29 (10.4%)	10 (16.4%)	1.774	0.183
Family history of early‐onset stroke	72 (25.8%)	8 (13.1%)	4.481	0.034
CI or TIA at enrollment	118 (42.6%)	28 (45.9%)	1.252	0.535
PCA involvement	123 (44.1%)	18 (29.5%)	4.383	0.036

Abbreviations: AS‐MMV, atherosclerosis‐associated moyamoya vasculopathy; CI, cerebral infarction; HRMRI, high‐resolution MRI; MMD, moyamoya disease; PCA, posterior cerebral artery. TIA, transient ischemic attack.

### 3.2 Associations Between HRMRI Characteristics and MMD Ischemic Hemispheres in Multivariable GEE Models

In the 279 MMD patients with 558 hemispheres, 169 hemispheres were classified as the ischemic group, while 389 hemispheres were categorized as the asymptomatic group. In multivariable GEE analyses adjusting for age, sex, and vascular risk factors, degree of MCA stenosis (OR = 1.438, 95% CI 1.132–1.828, *p* = 0.003), MCA remodeling index (OR = 0.675, 95% CI 0.479–0.950, *p* = 0.024), Suzuki stage (OR = 1.193, 95% CI 1.087–1.308, *p* < 0.001), ICA score (OR = 1.341, 95% CI 1.43–1.574, *p* < 0.001), MCA score (OR = 1.177, 95% CI 1.063–1.304, *p* = 0.002), and PCA score (OR = 2.493, 95% CI 1.806–3.441, *p* < 0.001) were all independently associated with ischemic hemispheres. MCA eccentricity index and ACA score did not show statistically significant associations (all *p* > 0.05) (Table 3, Figure 3).

**TABLE 3:** Associations between HRMRI characteristics and MMD ischemic hemispheres in multivariable GEE models adjusted for clinical risk factors.

	Asymptomatic (*n* = 389)	Ischemic (*n* = 169)	*p*	OR	95% CI	
Vessel Wall Image						
Degree of MCA stenosis	0.86 (0.79)	0.93 (0.36)	0.003	1.438	1.132	1.828
MCA eccentricity index	0.07 (0.24)	0.10 (0.26)	0.075	1.564	0.705	3.468
MCA remodeling index	0.47 (0.70)	0.47 (0.43)	0.024	0.675	0.479	0.950
Vascular Involvement						
Suzuki staging	3 (1)	4 (2)	< 0.001	1.193	1.087	1.308
ICA score	1 (1)	2 (1)	< 0.001	1.341	1.143	1.574
MCA score	2 (2)	3 (1)	0.002	1.177	1.063	1.304
ACA score	1 (2)	1 (1)	0.340	1.104	0.901	1.354
PCA score	0 (0)	1 (1)	< 0.001	2.493	1.806	3.441

Abbreviations: ACA, anterior cerebral artery; CI, Cconfidence Iinterval. GEE, generalized estimating equations; HRMRI, high‐resolution MRI; ICA, internal carotid artery; MCA, middle cerebral artery; MMD, moyamoya disease; OR, odds ratio; PCA, posterior cerebral artery.

**FIGURE 3:**
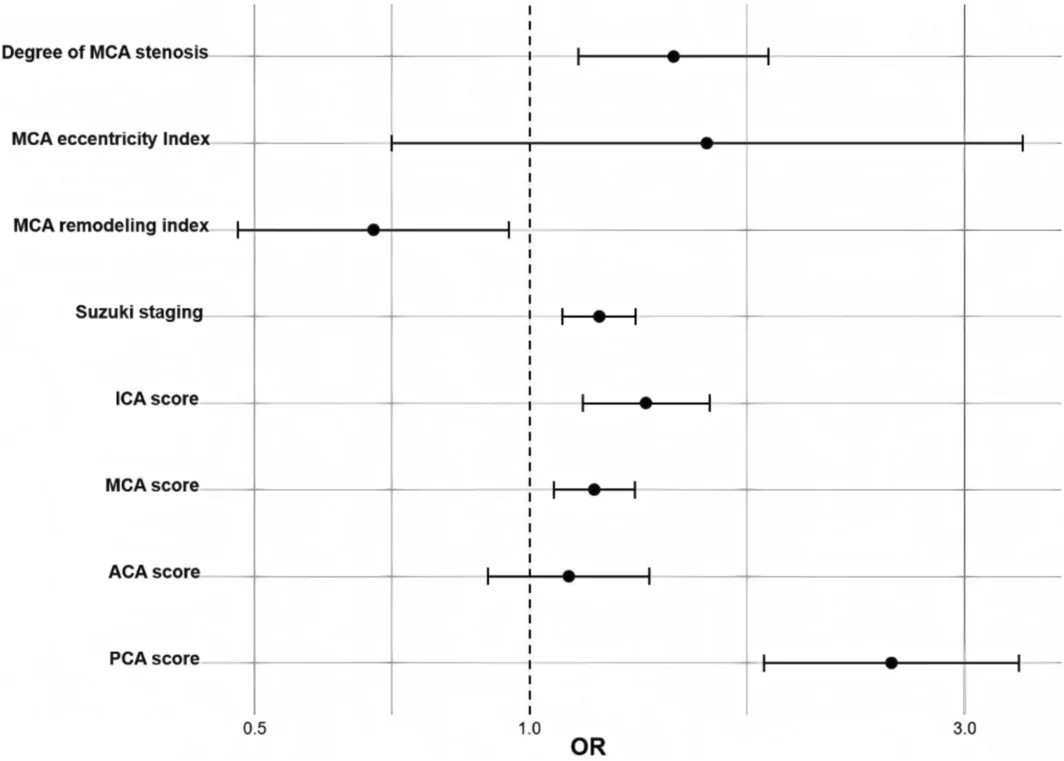
Adjusted associations between HRMRI characteristics and MMD ischemic hemispheres in GEE models.

### 3.3 Associations Between HRMRI Characteristics and AS‐MMV Ischemic Hemispheres in Multivariable GEE Models

In the 61 AS‐MMV patients with 122 hemispheres, 31 hemispheres were classified as the ischemic group, while 91 hemispheres were categorized as the asymptomatic group. In multivariable GEE analyses adjusting for age, sex, and vascular risk factors, both MCA remodeling index (OR = 3.322, 95% CI 1.631–6.767, *p* < 0.001) and ACA score (OR = 2.172, 95% CI 1.021–4.624, *p* = 0.044) were independently associated with ischemic hemispheres. Degree of MCA stenosis, MCA eccentricity index, Suzuki stage, ICA score, MCA score and PCA score did not show statistically significant associations (all *p* > 0.05) (Table 4, Figure 4). Besides, 24 of the 340 MMV patients in our cohort did not show ICA involvement on MRA (15 in the MMD group and 9 in the AS‐MMV group), but all had clear ACA and/or MCA stenosis or occlusion together with moyamoya collateral vascular networks. In a sensitivity analysis excluding 24 patients without ICA involvement, the associations between HRMRI measures and ischemic hemispheres in both the MMD and AS‐MMV subgroups remained qualitatively unchanged (shown in Table S4 and Table S5).

**TABLE 4:** Associations between HRMRI characteristics and AS‐MMV ischemic hemispheres in multivariable GEE models adjusted for clinical risk factors.

	Asymptomatic (*n* = 91)	Ischemic (*n* = 31)	*p*	OR	95% CI	
Vessel Wall Image						
Degree of MCA stenosis	0.64 (0.85)	0.68 (0.84)	0.087	0.629	0.370	1.069
MCA eccentricity index	0.62 (0.73)	0.69 (0.77)	0.745	1.051	0.779	1.419
MCA remodeling index	0.90 (0.42)	1.00 (0.22)	< 0.001	3.322	1.631	6.767
Vascular Involvement						
Suzuki staging	3 (1)	3 (2)	0.933	1.007	0.853	1.189
ICA score	1 (1)	1 (1)	0.766	1.079	0.655	1.777
MCA score	1 (1)	2 (2)	0.567	1.068	0.853	1.336
ACA score	0 (1)	1 (2)	0.044	2.172	1.021	4.624
PCA score	0 (0)	0 (1)	0.524	1.101	0.820	1.478

Abbreviations: ACA, anterior cerebral artery; AS‐MMV, atherosclerosis‐associated moyamoya vasculopathy; CI, confidence interval. GEE, generalized estimating equations; HRMRI, high‐resolution MRI; ICA, internal carotid artery; MCA, middle cerebral artery; OR, odds ratio; PCA, posterior cerebral artery.

**FIGURE 4:**
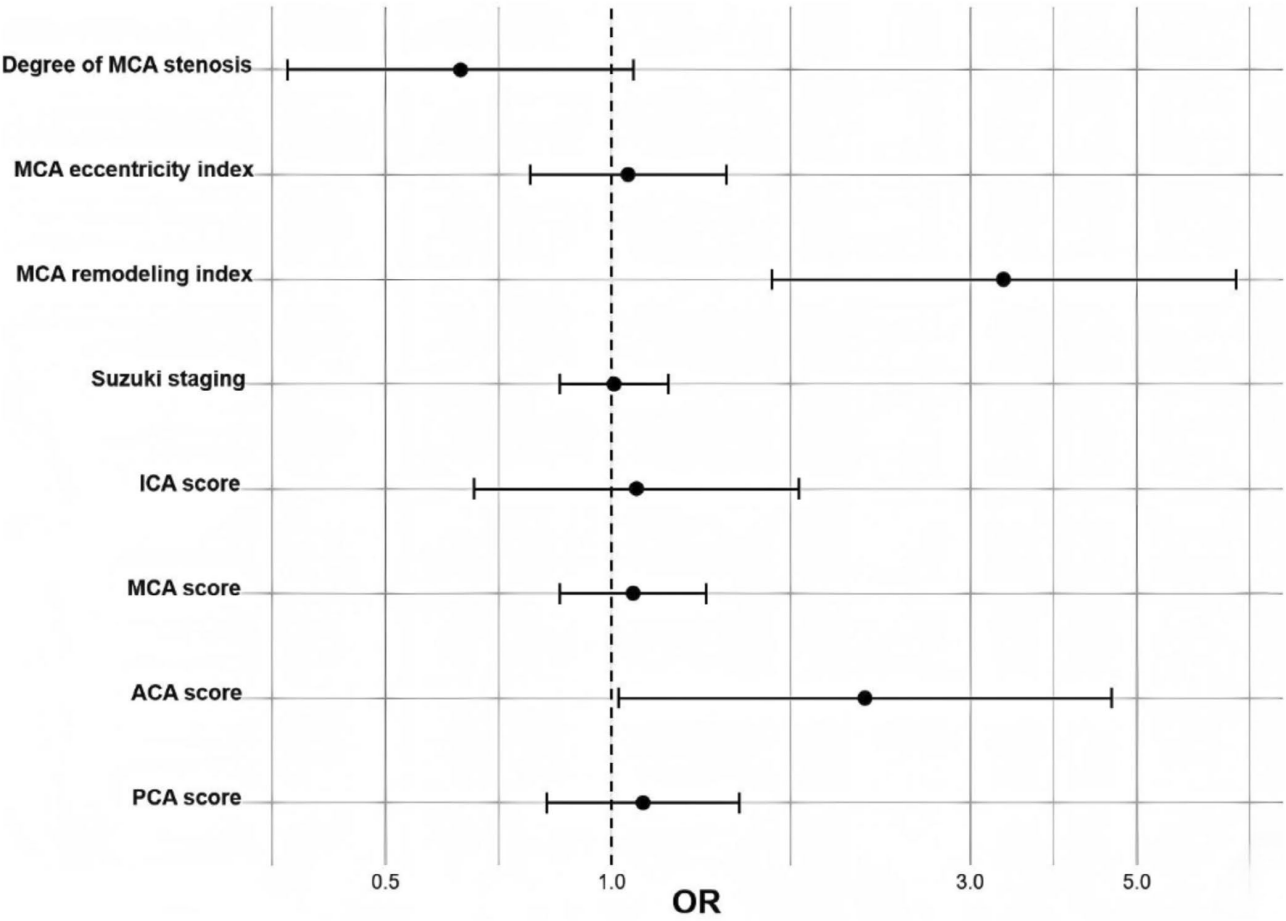
Adjusted associations between HRMRI characteristics and AS‐MMV ischemic hemispheres in GEE models.

## 4 Discussion

In our study, we categorized adult patients from the MMV cohort into MMD and AS‐MMV groups based on previously established HRMRI characteristics. The results revealed that MMD patients had a higher proportion of females and were more likely to have a family history of early‐onset stroke and PCA involvement. Subsequently we found, in MMD, the burden and extent of steno‐occlusive changes in the anterior and posterior circulations—as captured by MCA stenosis severity, Suzuki stage, and ICA/MCA/PCA scores—were independently associated with ischemic hemispheres, whereas a higher MCA remodeling index was protective. However, in AS‐MMV, ischemic hemispheres were instead characterized by an increased MCA remodeling index and ACA involvement, while the degree of MCA stenosis and other vascular involvement scores were not significantly associated with ischemia after adjustment. Together, these results suggest that the mechanisms linking HRMRI vascular phenotypes to ischemia differ substantially between MMD and AS‐MMV, highlighting fundamental differences in pathogenesis, intracranial vascular compensation patterns, and hemodynamic alterations between the two etiological subtypes of adult MMV, with significant implications for clinical management and therapeutic decision‐making.

In MMD patients, characteristic concentric thickening and negative remodeling were observed on HRMRI, consistent with the progressive intimal hyperplasia pathognomonic of MMD. In contrast, AS‐MMV patients typically exhibited eccentric plaque formation and positive remodeling patterns indicative of atherosclerotic pathology [5, 8]. These distinct HRMRI signatures enable more accurate differential diagnosis than conventional imaging modalities alone. The vessel wall imaging capabilities of HRMRI proved particularly valuable in identifying disease‐specific patterns in MMV. In this study, MMD patients were more frequently female than AS‐MMV patients, which is consistent with the established understanding that MMD is a disease with a female predominance [19]. Besides, the significant association between MMD and family history of early‐onset stroke and PCA involvement in our study underscores the genetic predisposition and more extensive cerebrovascular involvement characteristic of MMD. Previous genetic association studies have identified RNF213 as the principal susceptibility gene for MMD, with the single nucleotide polymorphism p.R4810K recognized as the founder variant predominantly in the Asian populations [20]. Wang et al. found that the RNF213 p.R4810K variant is associated with earlier disease onset and more severe PCA involvement in Chinese patients with MMD [21]. The observed genotype–phenotype correlations may potentially explain the differential manifestations between MMD and AS‐MMV in our study, particularly regarding family history of early‐onset stroke and PCA involvement. The clinical differences between MMD and AS‐MMV observed in our cohort are in line with the current concept that MMD is more strongly driven by genetic and familial factors, whereas AS‐MMV represents a secondary moyamoya‐like pattern superimposed on intracranial atherosclerosis. In the research by Han et al., however, moyamoya syndrome patients with plaques detected on HRMRI were predominantly elderly, with lower prevalence of cerebral hemorrhage and disease progression [4], which were not significantly reproduced in our current cohort. Therefore, we conclude that relying solely on the presence or absence of plaques is insufficient for definitive etiological differentiation of adult MMV, which may lead to misclassification of MMD cases with mild atherosclerotic burden. In clinical practice, comprehensive etiological assessment should rely primarily on HRMRI features and family history, with patient age and cerebrovascular risk factor profile considered as supportive rather than standalone indicators when evaluating the etiology of MMV.

Although previous studies have extensively investigated differences in vascular imaging features between asymptomatic and symptomatic MMV, such as choroidal anastomosis, PCA involvement, and anterior choroidal artery (AChA) dilatation, most were based on cerebral digital subtraction angiography, with relatively few studies utilizing noninvasive vascular imaging like HRMRI [11, 22, 23, 24]. Our study specifically focuses on the correlation between HRMRI‐based vessel wall parameters, vascular involvement severity, and the MCA territory‐related infarction in MMV. The hemispheric GEE analyses highlighted that, in MMD, ischemic hemispheres are primarily characterized by the overall severity and spatial extent of steno‐occlusive disease. Higher degrees of MCA stenosis, more advanced Suzuki stages, and higher ICA/MCA/PCA scores were all independently associated with ischemia, even after controlling for age, sex, and conventional vascular risk factors. This pattern underscores that ischemic events in MMD might be a consequence of progressive narrowing and occlusion of the major intracranial arteries together with poor collateral compensation, particularly from the posterior to the anterior circulation. Notably, the MCA remodeling index showed an inverse association with ischemic hemispheres in MMD, suggesting that more constrictive remodeling of the MCA wall may aggravate hemodynamic compromise in the setting of fixed luminal narrowing. In other words, for a given degree of stenosis in MMD patients, hemispheres with greater PCA compensatory and milder constrictive remodeling may preserve cerebral perfusion more effectively and thereby be less likely to present as the ischemic side.

In contrast, the ischemic pattern observed in AS‐MMV patients appeared to be associated with a different vascular involvement phenotype. In the AS‐MMV subgroup, it is noteworthy that the degree of MCA stenosis was not independently associated with MCA‐related ischemic hemispheres after adjustment, whereas the MCA remodeling index and ACA involvement remained significant. This finding likely reflects that, unlike idiopathic MMD, ischemia in AS‐MMV may be more strongly associated with how the vessel wall remodels around atherosclerotic plaques and the integrity of collateral pathways, rather than the percentage of luminal narrowing alone. Vascular positive remodeling represents a significant imaging characteristic of intracranial atherosclerotic disease (ICAD) on HRMRI [25, 26]. The study by Guo et al. revealed that culprit plaques identified based on cerebral ischemic infarcts demonstrated greater longitudinal luminal expansion on HRMRI [27], highlighting the significant association between vascular remodeling index and both atherosclerotic burden and cerebral infarction risk. In our study, the positive association between MCA remodeling index and ischemic hemispheres in AS‐MMV may reflect a more “atherosclerotic” remodeling behavior, in which positive and eccentric remodeling accompanies plaque growth and vulnerability rather than protective compensation. The remodeling index may also capture a larger proportion of the variance linked to ischemia and attenuate the apparent effect of MCA stenosis in multivariable models. At the same time, the significance of ACA involvement highlights differences in poststenotic intracranial vascular compensation patterns compared to MMD. Our study demonstrated the ACA in AS‐MMV frequently may act as a key source of collateral flow to mitigate cerebral ischemia, whereas its role in MMD is often less prominent due to the disease's diffuse nature affecting the entire circle of Willis [15]. The lack of a strong association between PCA involvement and ischemia in AS‐MMV, in contrast to MMD, also underscores the distinct vascular territories that drive ischemic risk in the two subtypes.

These divergent patterns have important potential clinical implications. They support the view that MMD and AS‐MMV should not be treated as a single homogeneous entity in research or clinical decision‐making [5, 6, 18]. In MMD, quantitative indices such as the degree of MCA stenosis, Suzuki stage, and combined ICA/MCA/PCA scores, together with reduced MCA remodeling, may help identify hemispheres at particularly high ischemic risk, potentially informing surgical timing and the selection of target hemispheres for revascularization. In AS‐MMV, however, a focus on MCA remodeling behavior and ACA involvement may be more relevant than simple stenosis severity, aligning the evaluation more closely with plaque characteristics and the integrity of anterior circulation collaterals.

Our study also has important limitations that should be acknowledged. Firstly, the design was retrospective and single‐center, which may introduce selection bias and limit generalizability to other populations or imaging protocols. Secondly, our study specifically addressed MCA territory‐related infarction rather than all possible patterns of infarct distribution. In addition, the AS‐MMV subgroup was relatively small, resulting in a limited number of ischemic hemispheres and some large ORs with wide CIs, particularly for the remodeling index. Although GEE models accounted for intrapatient correlation between hemispheres, residual confounding by unmeasured factors (such as genetic variants, hemodynamic parameters, or plaque composition) cannot be excluded. Fourth, 7.1% of 340 patients did not show ICA involvement on MRA, although all had clear ACA and/or MCA disease with typical collaterals. It is possible that some of these cases partially overlap with chronic focal cerebral arteriopathy or other mimics, and this may limit the generalizability of our results to populations defined strictly by the updated diagnostic criteria requiring ICA involvement. Another limitation is that our definition of ischemic hemispheres included both acute and chronically infarcted territories. The HRMRI and MRA patterns observed in chronically ischemic hemispheres may not fully reflect the vessel status at the time the ischemic event occurred, and they cannot be directly interpreted as indicators of active, progressive disease, or predictors of future ischemic risk. Our findings should therefore be viewed as cross‐sectional associations between vessel wall and luminal characteristics and the presence of ischemia, rather than as causal relationships or prognostic markers.

Despite these limitations, this study provides a detailed, hemisphere‐level characterization of HRMRI vascular features in a relatively large cohort of MMV patients, explicitly separating MMD from AS‐MMV and applying cluster‐adjusted GEE models to account for within‐patient correlation. The finding that MMD ischemic hemispheres are characterized by global steno‐occlusive burden and negative vascular remodeling, whereas AS‐MMV ischemic hemispheres are more closely related to positive vascular remodeling and ACA involvement, offers a pathophysiologically plausible framework that aligns with the distinct etiologies of these two entities. The recent 7‐year follow‐up results from Jiao et al. on surgical treatment for intracranial arterial stenosis demonstrated no significant benefit from stenting, while revascularization may represent a viable therapeutic option for AS‐MMV [28]. Therefore, applying appropriate and informative assessment strategies to guide the timing and indications of revascularization in patients with MMV is crucial for optimizing their prognosis. Future prospective, multicenter studies incorporating hemodynamic assessments, longitudinal follow‐up, and surgical outcomes will be helpful to validate these imaging markers and determine whether they can be integrated into risk stratification and treatment decision algorithms for patients with MMV.

## Author Contributions

Guangsong Han and Jiachun Pan contributed to the analysis and interpretation of the data, the study design and the writing of the manuscript. Yuehui Hong and Xiaoyuan Fan contributed to the analysis and interpretation of the data. Ming Yao, Lixin Zhou, Yicheng Zhu, and Feng Feng contributed to the collection and interpretation of data. Jun Ni contributed to the study design and reviewing of the manuscript. All coauthors have read and approved the final manuscript.

## Funding

This study was supported by a grant from the National Natural Science Foundation of China (No. 82571516) and National High‐Level Hospital Clinical Research Funding (2022‐PUMCH‐D‐007).

## Ethics Statement

Written informed consent was obtained from all subjects (patients) in this study. The study conformed with World Medical Association Declaration of Helsinki published on the website of the Journal of American Medical Association. Institutional Review Board approval was obtained (ethics number: ZS‐2926).

## Conflicts of Interest

The authors declare no conflicts of interest.

## Supporting information


**Table S1:** The parameters for the 3.0 T MRI scanner.
**Table S2:** The Suzuki staging system.
**Table S3:** Inter‐Rater Agreement of Imaging Data.
**Table S4:** Sensitivity analysis of associations between HRMRI characteristics and ischemic hemispheres in MMD after excluding patients without ICA involvement.
**Table S5:** Sensitivity analysis of associations between HRMRI characteristics and ischemic hemispheres in AS‐MMV after excluding patients without ICA involvement.

## Data Availability

The data that support the findings of this study are available from the corresponding author upon reasonable request.
