# Supplementary material for: High‐Resolution MRI Revealed Different Etiology‐Specific Associations With Cerebral Infarction in Adult Moyamoya Vasculopathy
Source: Ann Clin Transl Neurol. 2026 Apr 1:10.1002/acn3.70380. Online ahead of print. doi: 10.1002/acn3.70380 (PMC13394470; doi:10.1002/acn3.70380)
Supplement: Supplementary file 1 — Table S1: The parameters for the 3.0 T MRI scanner. Table S2: The Suzuki staging system. Table S3: Inter‐Rater Agreement of Imaging Data. Table S4: Sensitivity analysis of associations between HRMRI characteristics and ischemic hemispheres in MMD after excluding patients without ICA involvement. Table S5: Sensitivity analysis of associations between HRMRI characteristics and ischemic hemispheres in AS‐MMV after excluding patients without ICA involvement. [file ACN3-9999-0-s001.docx]

Supplementary Table 1 The parameters for the 3.0T MRI scanner

|  | TR(ms) | TE(ms) | TI(ms) | FA(°) | slice thickness  (mm) | number of slices | acquisition matrix | FOV | B_max_ (s/mm^2^) |
| --- | --- | --- | --- | --- | --- | --- | --- | --- | --- |
| T1WI | 1775 | 21.6 | 720 | 111 | 4 | 40 | 320×160 | 240×168 | NA |
| T2WI | 5258 | 84 | NA | 142 | 4 | 36 | 320×320 | 220×220 | NA |
| FLAIR | 12000 | 122 | 2712 | 160 | 4 | 36 | 288×192 | 220×176 | NA |
| DWI | 3600 | 64 | NA | 90 | 4 | 36 | 128×128 | 220×220 | 1000 |
| TOF-MRA | 16 | 2.1 | NA | 20 | 1.2 | 288 | 256×256 | 220×220 | NA |
| HRMRI | 800 | 15.8 | NA | 12 | 0.8 | 248 | 320×256 | 204×184 | NA |

TR: Repetition time; TE: echo time; TI: inversion time; FA: flip angle; FOV: field of view; T1WI: T1-weighted imaging; T2WI: T2-weighted imaging; FLAIR: fluid-attenuated inversion recovery; DWI: diffusion-weighted imaging; TOF-MRA: time-of-flight MR angiography; HRMRI: high-resolution MR.

Supplementary Table 2 The Suzuki staging system

| Stage | Findings |
| --- | --- |
| I | Narrowing of the carotid fork |
| II | Initiation of the moyamoya with dilation of the intracerebral main arteries |
| III | Intensification of the moyamoya with partial disappearance of the intracerebral main arteries |
| IV | Minimization of the moyamoya, advanced steno-occlusive internal carotid artery, ACA, and MCA with gradually enlarged collateral from the extracranial area |
| V | Reduction of the moyamoya, disappearance of ACA and MCA, with increased collateral from the external carotid artery |
| VI | Disappearance of the moyamoya, with cerebral circulation maintained only by the external carotid artery or the vertebral artery |

ACA: anterior cerebral artery; MCA: middle cerebral artery.

Supplementary Table 3 Inter-Rater Agreement of Imaging Data

|  | Intraclass Correlation Coefficient | 95% Confidence Interval | | P |
| --- | --- | --- | --- | --- |
| Degree of MCA stenosis | 0.764 | 0.621 | 0.846 | 0.035 |
| MCA eccentricity index | 0.805 | 0.746 | 0.869 | 0.013 |
| MCA remodeling index | 0.796 | 0.725 | 0.842 | 0.024 |
| Suzuki staging | 0.969 | 0.943 | 0.983 | ＜0.001 |
| ICA score | 0.928 | 0.907 | 0.949 | ＜0.001 |
| MCA score | 0.878 | 0.841 | 0.914 | ＜0.001 |
| ACA score | 0.901 | 0.854 | 0.938 | ＜0.001 |
| PCA score | 0.884 | 0.867 | 0.919 | ＜0.001 |

ICA: internal carotid artery; MCA: middle cerebral artery; ACA: anterior cerebral artery; PCA: posterior cerebral artery.

Supplementary table 4 Sensitivity analysis of associations between HRMRI characteristics and ischemic hemispheres in MMD after excluding patients without ICA involvement

|  | P | OR | 95% CI | |
| --- | --- | --- | --- | --- |
| Vessel Wall Image |  |  |  |  |
| Degree of MCA stenosis | **0.021** | 1.271 | 1.036 | 1.559 |
| MCA eccentricity index | 0.290 | 1.525 | 0.698 | 3.329 |
| MCA remodeling index | **0.005** | 0.640 | 0.467 | 0.875 |
| Vascular Involvement |  |  |  |  |
| Suzuki staging | **0.004** | 1.138 | 1.042 | 1.243 |
| ICA score | **0.002** | 1.289 | 1.094 | 1.520 |
| MCA score | **0.015** | 1.114 | 1.021 | 1.216 |
| ACA score | 0.646 | 1.050 | 0.853 | 1.291 |
| PCA score | **＜0.001** | 2.230 | 1.629 | 3.053 |

HRMRI: high-resolution MRI; MMD: moyamoya disease; GEE: generalized estimating equations; ICA: internal carotid artery; MCA: middle cerebral artery; ACA: anterior cerebral artery; PCA: posterior cerebral artery; OR: odds ratio; CI: Confidence Interval.

Supplementary table 5 Sensitivity analysis of associations between HRMRI characteristics and ischemic hemispheres in AS-MMV after excluding patients without ICA involvement

|  | P | OR | 95% CI | |
| --- | --- | --- | --- | --- |
| Vessel Wall Image |  |  |  |  |
| Degree of MCA stenosis | 0.124 | 0.620 | 0.337 | 1.141 |
| MCA eccentricity index | 0.679 | 1.076 | 0.760 | 1.524 |
| MCA remodeling index | **0.001** | 2.815 | 1.486 | 5.332 |
| Vascular Involvement |  |  |  |  |
| Suzuki staging | 0.940 | 1.008 | 0.824 | 1.233 |
| ICA score | 0.846 | 1.047 | 0.656 | 1.672 |
| MCA score | 0.532 | 1.081 | 0.846 | 1.381 |
| ACA score | **0.040** | 2.142 | 1.036 | 4.430 |
| PCA score | 0.163 | 1.217 | 0.923 | 1.605 |

HRMRI: high-resolution MRI; AS-MMV: atherosclerosis-associated moyamoya vasculopathy; GEE: generalized estimating equations; ICA: internal carotid artery; MCA: middle cerebral artery; ACA: anterior cerebral artery; PCA: posterior cerebral artery; OR: odds ratio; CI: Confidence Interval.
